# Selective Antiproliferative and Apoptotic Effects of 2,6‐Diketopiperazines on MDA‐MB‐231 Triple‐Negative Breast Cancer

**DOI:** 10.1111/cbdd.70098

**Published:** 2025-04-02

**Authors:** Flor Paulina Garrido González, Martha Edith Macías Pérez, Octavio Rodríguez Cortés, Elvia Mera Jiménez, Teresa Mancilla Percino

**Affiliations:** ^1^ Centro de Investigación y de Estudios Avanzados del Instituto Politécnico Nacional, Departamento de Química Ciudad de México México; ^2^ Instituto Politécnico Nacional, Sección de Estudios de Posgrado e Investigación, Escuela, Superior de Medicina, Plan de San Luis y Diaz Mirón Ciudad de México México

**Keywords:** 2,6‐diketopiperazines, anticancer agents, apoptosis, biological activity, cytotoxicity

## Abstract

Diketopiperazines (DKPs) have emerged as promising candidates for treating diverse diseases, particularly cancer. In this context, 2,5‐diketopiperazines have been extensively investigated in comparison with 2,6‐diketopiperazines. This work explores the selectivity and impact of 2,6‐diketopiperazine enantiomers derived from α‐amino acids on MDA‐MB‐231 triple‐negative breast cancer cells (TNBC). This subtype of cancer is recognized for its aggressiveness and the lack of effective therapeutic options. The evaluation utilized MTT and flow cytometry assays to examine the impact of 2,6‐DPKs on the MDA‐MB‐231 breast cancer cell line. The obtained IC_50_ values for compounds **1** and (*S*)‐enantiomers ranged from 4.6 μM to 4.944 mM, where **1** and (*S*)‐**2a** exhibited the lowest values. The IC_50_ values for (*R*) enantiomers ranged from 0.021 to 3.639 mM, whereas (*R*)‐**2b** was the lowest. Flow cytometry results revealed that compounds increase in apoptosis at 48 h compared to 24 h, ranging from 54.1% to 76.2%; however, (*S*)‐**12a** exhibited a 3% decrease in apoptotic induction at 48 h. All investigated 2,6‐diketopiperazines derived from α‐amino acids showed potential as anticancer agents against MDA‐MB‐231 cancer cells. In particular, compounds **1**, (*S*)‐**2a**, ‐4a, and ‐**5a** showed remarkable inhibitory effects on proliferation, viability, and apoptosis. The MTT results in the culture of healthy cells of the kidney line Vero did not display cytotoxicity at the tested concentrations up to 12.0 mM. Hence, these 2,6‐DPKs are suitable for in vivo testing shortly.

## Graphical abstract

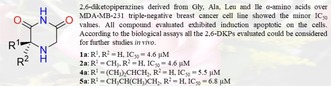

## 1 Introduction

Piperazinediones, the smallest cyclic peptides, possess a unique structure characterized by a piperazine ring and two carbonyl groups, resulting in three isomers known as 2,3‐, 2,5‐ and 2,6‐Diketopiperazines (DKPs) (Fischer 2003). These heterocycles compounds and their derivatives have garnered significant attention due to their presence in numerous natural products (Borthwick 2012; Huang et al. 2014; Al‐Khdhairawi et al. 2023; Chen et al. 2022), and their diverse range of biological properties, including antihyperglycemic (Martins and Carvalho 2007), antibacterial (Martins and Carvalho 2007; Wolf‐Rainer 2005; Buroni et al. 2018), anticancer (Al‐Khdhairawi et al. 2023; Martins and Carvalho 2007; Shvedaite and Shimkyavichene 1999; Kim and Movassaghi 2015; Wang et al. 2023) and neuroprotective effects agents (Cornacchia et al. 2012), as well as their potential as oxytocin antagonists (Yang et al. 2019) and influenza virus inhibitors (Wu et al. 2017). Additionally, DKPs have demonstrated affinity to calcium channels, σ1 and σ2 receptors and anti‐neoplastic activity (Kilian et al. 2005), and sigma receptor binders (Ghandi et al. 2016), while displaying anticonvulsant (Wei et al. 2015; Dawidowski et al. 2011), cytotoxic (Liao et al. 2016), and trypanocidal activities (Jones et al. 2013; Fytas et al. 2019), among others. Although the 2,6‐DKPs isomers have received comparatively less attention, recent synthetic strategies have aimed to provide facile access to the imide moiety, which is responsible for the observed biological properties (Mancilla et al. 2002; Dinsmore and Beshore 2002; Garrido González and Mancilla Percino 2020; González‐Saiz et al. 2022). Notably, the bis(2,6‐DKP) framework is present in several compounds, such as ICRF‐154 and its derivatives (ICRF‐159, ICRF‐187, ICRF‐193, and MST‐16), which have shown promising inhibition of the topoisomerase II enzyme, an essential enzyme involved in DNA replication and transcription (Nitiss 2009; Tanabe et al. 1991). Furthermore, DKPs have demonstrated significant antitumor activity against several types of cancers, including Lewis lung carcinoma, sarcoma 180 and S‐37, L1210 and P388 leukemia, B16 melanoma, malignant lymphoma, C‐26 colon adenocarcinoma, and C‐38 human colon cancer (Andoh and Ishida 1998; Narita et al. 1991; Ren et al. 1985; Lu and Lu 2010). ICRF derivatives reached clinical trials (Narita et al. 1991; Langer 2014; Wheeler et al. 1983), among which MST‐16 was particularly efficacious in treating adult *T*‐cell leukemia and non‐Hodgkin's lymphoma (Ohno et al. 1993; Okamoto et al. 2002). Our research group has focused on the synthesis of 2,6‐DKPs derived from α‐amino acids (Mancilla et al. 2002), and docking studies on hTopo IIα topoisomerase inhibitors, where the binding modes depended on the stereochemistry (Correa et al. 2012). Recently, we have reported the docking study, synthesis, and inhibitory activity on HDAC8 of 2,6‐DKPs derived from α‐amino acids (Garrido González and Mancilla Percino 2020), those results suggested that the compounds may be promising anticancer agents for further biological studies. It has been described that overexpression of histone deacetylase 8 (HDAC8) is linked with several types of cancer, including colon, breast, lung, childhood neuroblastoma, and hematological malignancies (Chakrabarti et al. 2016; Amin et al. 2023), and knockdown of HDAC8 has shown to inhibit the proliferation and viability of cancer cells (Banerjee et al. 2019) reduction of clonogenic growth, cell cycle arrest and induction of cell differentiation without affecting global histone acetylation or cellular HDAC activity (Witt et al. 2009). According to Global Cancer (GLOBOCAN 2020) breast cancer (BC) is the most common cancer, reporting 11.7% of all cancers, and around 685,000 women died in 2020 due to the disease (Globocan 2020). Among the subclasses of BC is triple‐negative breast cancer (TNBC), which accounts for 10%–20% of BC, TNBC is characterized by the absence of estrogen (ER), progesterone (PR), and Her2/neu receptors and is among the most aggressive tumors yet without effective therapies (Dent et al. 2007). It has been shown that HDAC isoforms participate in the invasion and/or invasion and/or migration in different TNBC cell lines. For instance, there was found increase in HDAC8 gene expression for the MDA‐MB‐231 cell line, while the MCF‐7 cell line showed higher gene expression of HDAC 4 and 6 (Park et al. 2011). On the other hand, cell death is an important event in maintaining the organism's homeostasis; among the most studied types of cell death is apoptosis. Apoptosis, in contrast to other types of cell death such as necrosis, pyroptosis, or NETosis, may occur in response to cellular stress, infection, DNA damage, or radiation, and is non‐inflammatory cell death (Davidovich et al. 2014). Apoptosis and necrosis represent two extensively documented forms of cell death, as revealed in multiple investigations involving HDAC inhibitors (iHDACs) (Galluzzi et al. 2018; Avi and Salvesen 2014). In this study, we extend the investigation into the biological activity of 2,6‐diketopiperazines (2,6‐DKPs), focusing specifically on their antiproliferative and inhibitory effects on cell viability. Our research explores the impact of compounds **1**, (*S*)‐(**2**–**14**), and (*R*)‐(**2**–**4**, **6**–**8**, and **11**–**14**) on the MDA‐MB‐231 breast cancer cell line. Additionally, we investigate the determination of cell death using flow cytometry for compounds **1**, **2a**‐**6a**, and **9a**‐**14a**. To determine the cytotoxicity of the most promising compounds **1**, (*S*)‐**2a**, −**4a**, and ‐**5a** in healthy cells, MTT assays were performed on cultures of the healthy kidney cell line Vero, which were incubated with gradually increasing concentrations from 0.00066 to12 mM.

## 2 Results and Discussion

**FIGURE 1:**
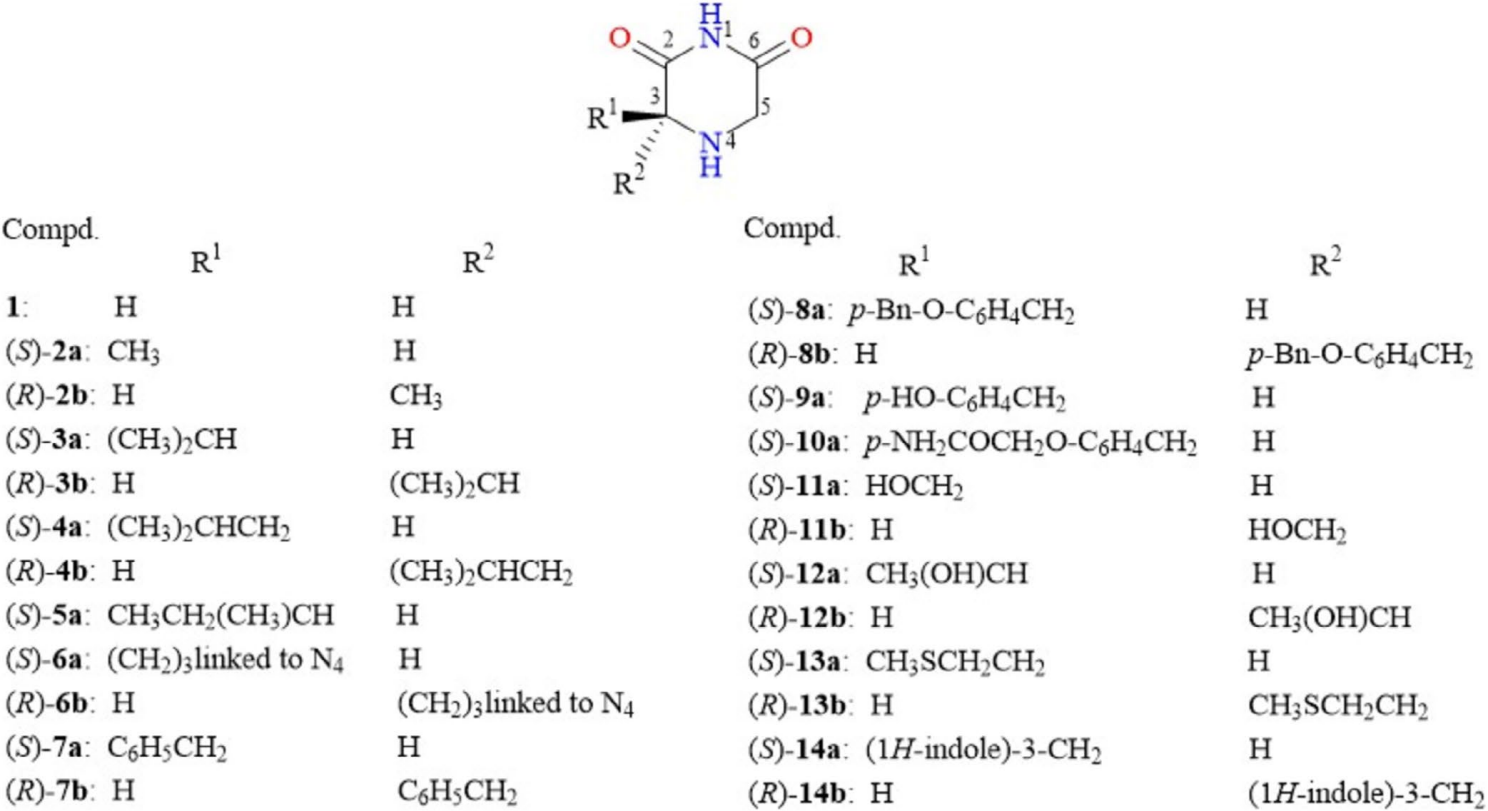
2,6‐DKPs derived from α‐amino acids assayed on MDA‐MB‐231.

This work is focused on investigating the impact of the various 2,6‐diketopiperazines (DKPs) derived from α‐amino acids on the MDA‐MB‐231 breast cancer line and their potential for apoptotic cell death induction through flow cytometry. The chemical structures of the tested 2,6‐DKPs were derived from Gly (**1**), stereoisomers derived from Ala (**2a**, **2b**), Val (**3a**, **3b**), Leu (**4a**, **4b**), Ile (**5a**), Pro (**6a**, **6b**), Phe (**7a**, **7b**), Tyr (**8a**, **8b**, with OH group protected by a Bn group), Tyr (**9a**), and Tyr (**10a**, with OH group protected by an acetamide group), Ser (**11a**, **11b**), Thr (**12a**, **12b**), Met (**13a**, **13b**), and Trp (**14a**, **14b**) as shown in Figure 1. All tested compounds were previously synthesized, characterized, and recently reported in the literature (Garrido González and Mancilla Percino 2020).

### 2.1 Antiproliferative Activity of 2,6‐DKPs on MDA‐MB‐231 Cell Line

The antiproliferative activity of 2,6‐diketopiperazines **1**, stereoisomers (*S*)‐**2a**‐**14a**, and (*R*)‐**2b**‐**4b**, **6b**‐**8b**, and **11b**‐**14b** was evaluated by the MTT assay on the MDA‐MB‐231 cell line using TSA as positive control at 50 μM. The study provided IC_50_ values given in Table 1. It was observed that 2,6‐DKPs efficacy varied significantly, with some exhibiting notably lower IC_50_ values. Compounds **1** and both (*S*) and (*R*) enantiomers reveal differences in their inhibitory effects. 2,6‐DKPs containing alkyl groups at the chiral center displayed enhanced inhibitory effects, specifically by (*S*)‐enantiomers.

**TABLE 1:** IC_50_ ^a^ of inhibitory activity of compound **1** and stereoisomers **2–14** on MDA‐MB‐231 triple‐negative breast cancer cells.

Compound	IC_50_	Compound	IC_50_
**1**	4.6 ± 0.019 μM		
(*S*)‐**2a**	4.6 ± 0.169 μM	(*R*)‐**2b**	0.021 ± 0.677 mM
(*S*)‐**3a**	0.017 ± 0.230 mM	(*R*)‐**3b**	0.822 ± 1.353 mM
(*S*)‐**4a**	5.5 ± 0.235 μM	(*R*)‐**4b**	0.265 ± 1.256 mM
(*S*)‐**5a**	6.8 ± 0.028 μM		
(*S*)‐**6a**	1.021 ± 0.167 mM	(*R*)‐**6b**	0.584 ± 1.007 mM
(*S*)‐**7a**	0.155 ± 0.322 mM	(*R*)‐**7b**	2.146 ± 0.202 mM
(*S*)‐**8a**	4.944 ± 0.733 mM	(*R*)‐**8b**	3.639 ± 1.218 mM
(*S*)‐**9a**	1.669 ± 0.011 mM		
(*S*)‐**10a**	0.469 ± 0.860 mM		
(*S*)‐**11a**	0.049 ± 0.026 mM	(*R*)‐**11b**	0.801 ± 0.208 mM
(*S*)‐**12a**	2.103 ± 0.249 mM	(*R*)‐**12b**	1.716 ± 0.085 mM
(*S*)‐**13a**	0.978 ± 0.096 mM	(*R*)‐**13b**	1.310 ± 0.284 mM
(*S*)‐**14a**	0.012 ± 0.452 mM	(*R*)‐**14b**	0.357 ± 0.190 mM

^a^ Results are the mean ± standard deviation of three separate determinations.

### 2.2 Apoptotic Induction of 2,6‐Diketopiperazines on MDA‐MB‐231 Cell Line

**FIGURE 2:**
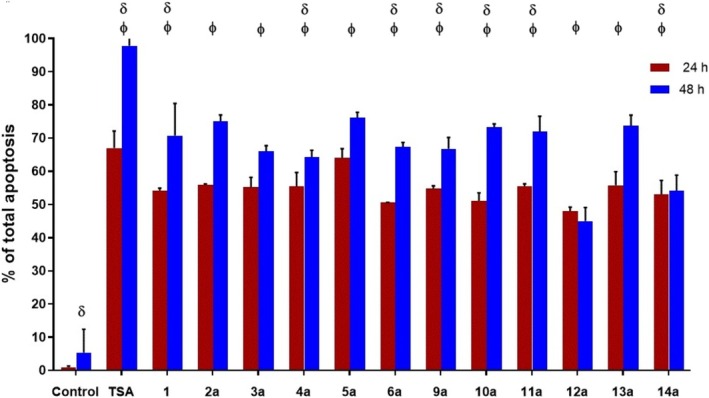
Percentage of total apoptotic cells of the MDA‐MB‐231 cell line treated with 1% DMSO (negative control), 50 μM TSA (positive control), and with the IC_50_ values of 2,6‐DKPs **1**, **2a**‐**6a**, and **9a**‐**14a** at 24 and 48 h. One‐way ANOVA. *p* < 0.05 between 24 h versus 48 h. **∏** *p* < 0.05 between compound vs. control.

The study proceeded to investigate the potential of the 2,6‐DKPs to induce apoptotic; both (*S*)‐ and (*R*)‐stereoisomers were examined. The findings indicated a higher rate of apoptosis induction at 48 h compared to 24 h, as shown in Figure 2. IC_50_ cell population charts are found in the Supporting Information SI. A minimal percentage of cell death occurred by necrosis. This highlighted the preference for apoptotic cell death induction, as shown in Figure S1 file.

**FIGURE 3:**
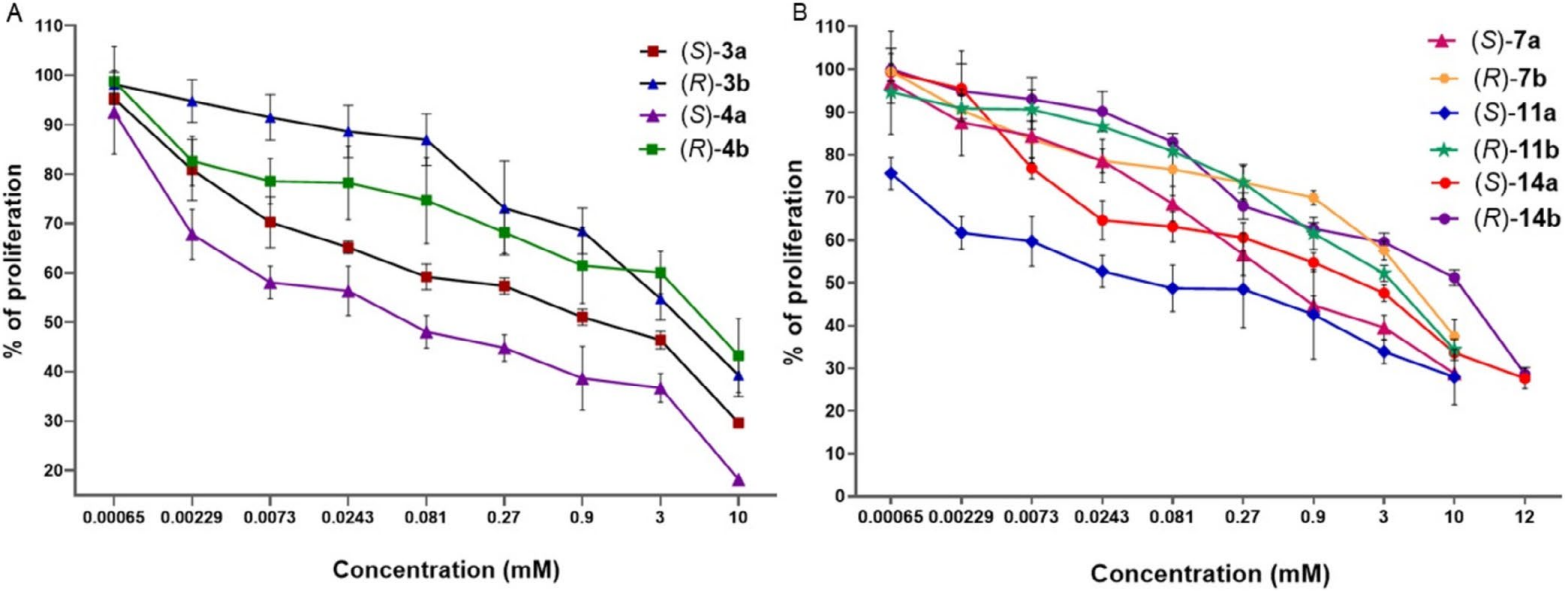
Concentration‐response curves for the 2,6‐DKPs enantiomeric (*S*)‐ and (*R*)‐pairs, (A) for **3** and **4** derived from Val and Leu α‐amino acids, respectively and (B) for **7, 11**, and **14** derived from Phe, Ser and Trp α‐amino acids, respectively, where (*S*)‐stereoisomers shown to be more potent than their corresponding (*R*) over the MDA‐MB‐231 cell line, *n* = 8, *p* < 0.0001.

Diketopiperazines derived from natural and unnatural amino acids are the smallest cyclic peptides, and due to their conformation, stereochemistry, and the type of substituent on the chiral center, they can bind to a large variety of receptors, giving a broad range of biological activities. Thus, they can give rise to a collection of compounds that may contribute to an understanding of the structural requirements for receptor interactions, allowing the validation of molecular targets and opening new perspectives for drug discovery (Martins and Carvalho 2007; Dawidowski et al. 2011; Garrido González and Mancilla Percino 2020). Analysis of IC_50_ values of **2a**, **3a**, **4a**, **7a**, **11a**, and **13a** showed them to be more potent than their corresponding (*R*) stereoisomers, while (*R*) stereoisomers **6b**, **8b**, and **12b** were shown to be slightly more potent than their corresponding (*S*) enantiomers. In addition, we also found that compounds had different ranges of IC_50_ values independently of their stereochemistry and the type of substituent on the chiral center as follows: for **2b**, **3a**, **11a**, and **14a**, they were in the range of 0.012 and 0.049 mM; in the range of 0.155 and 0.978 mM for **3b**, **4b**, **7a**, **10a**, **11b**, **13a**, and **14b**; between 1.021–1.716 mM for **6a**, **9a**, **12b**, and **13b**; for **7b** and **12a**, the values were 2.146 and 2.103 mM, respectively, and for **8a** and **8b**, they were 4.944 and 3.639 mM, respectively. These results suggest that not only the stereochemistry and the type of substituent on the chiral center influence the inhibitory effect on MDA‐MB‐231 triple‐negative breast cancer cells, but may also be due to the adopted conformations of the compounds under study. Figure 3A,B show the concentration‐response curves of the (*S*) and (*R*) enantiomeric pairs of **3**, **4** and **7**, **11**, and **14**, respectively, where the (*S*)‐stereoisomers shown to be more efficient than their corresponding (*R*) to inhibit the MDA‐MB‐231 cells.

**FIGURE 4:**
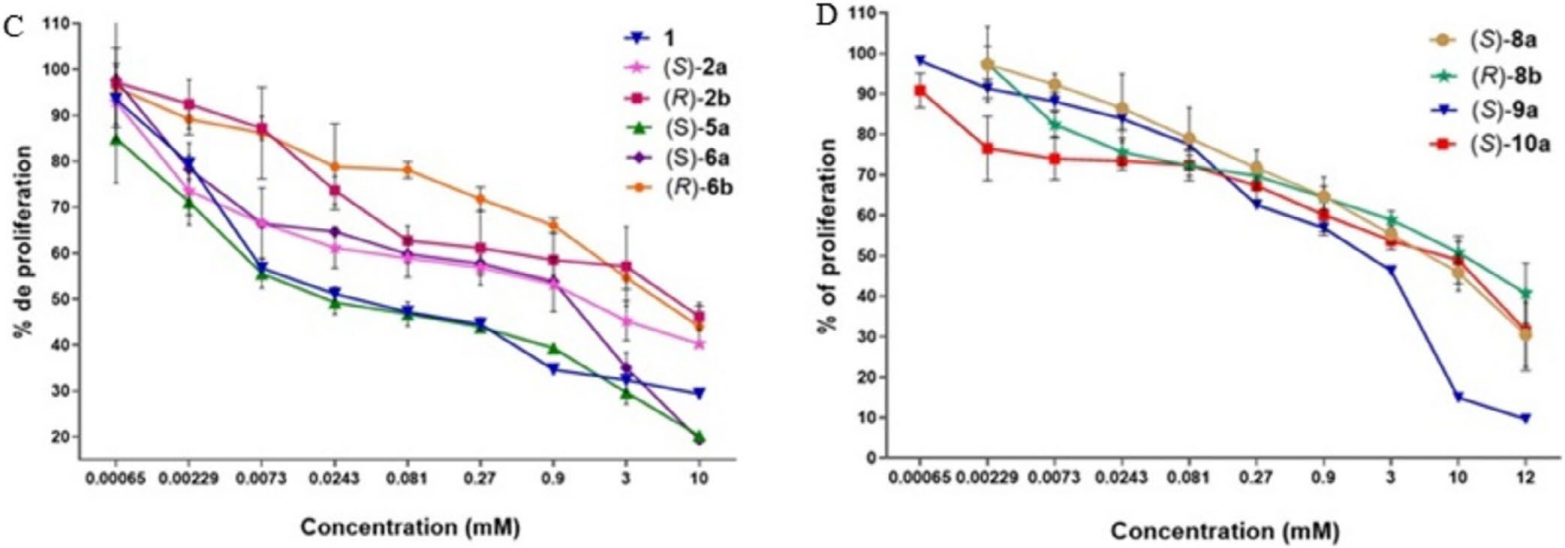
(C) Concentration‐response curves for the 2,6‐DKPs **1** (Gly derived), and stereoisomers **2a**, **2b** (Ala derivatives), **5a** (Ile derived), and **6a**, **6b** (Pro derivatives), (D) Concentration‐response curves for 2,6‐DKPs **8a**, **8b**, **9a** and **10a** stereoisomers derived of Tyr α‐amino acids on the MDA‐MB‐231 cell line, *n* = 8, *p* < 0.0001.

Figure 4C shows the concentration‐response curves for **1** (Gly derived) and stereoisomers **2a**, **2b** (Ala derivatives), **5a** (Ile derived), and **6a**, **6b** (Pro derivatives), where **1** and **5a** exhibited more potency than the others, and between pairs of enantiomers, the (*S*)‐enantiomers shown to be more potent inhibitors than their corresponding (*R*) on the MDA‐MB‐231 cell line. Concentration‐response curves for 2,6‐DKPs **8a**, **8b**, **9a**, and **10a** stereoisomers derived from Tyr α‐amino acids on the MDA‐MB‐231 cell line are in Figure 4D; **9a** was shown to be more potent than the stereoisomers **8a** and **8b (**with OH group protected by a Bn group) and **10a** (Tyr derived with OH group protected with an acetamide group).

Figure S2 shows concentration‐response curves for 2,6‐DKPs enantiomeric (*S*)‐ and (*R*)‐pairs **12** and **13** derived from Thr and Met α‐amino acids, respectively, over the MDA‐MB‐231 cell line, which data showed that (*S*) stereoisomers **12a** and **13a** are more potent than their corresponding (*R*) enantiomers.

Compounds **1**, (*S*)‐**2a**, −**4a**, and ‐**5a** presented the lowest IC_50_ values of all the compounds tested (see Table 1), so it was interesting to investigate their toxicity in healthy cells. Thus, these assays were carried out on the Vero kidney cell line, whose compounds showed 100% viability even at 12 mM concentrations (data not shown). The results suggest that these compounds show promise as anticancer agents.

**FIGURE 5:**
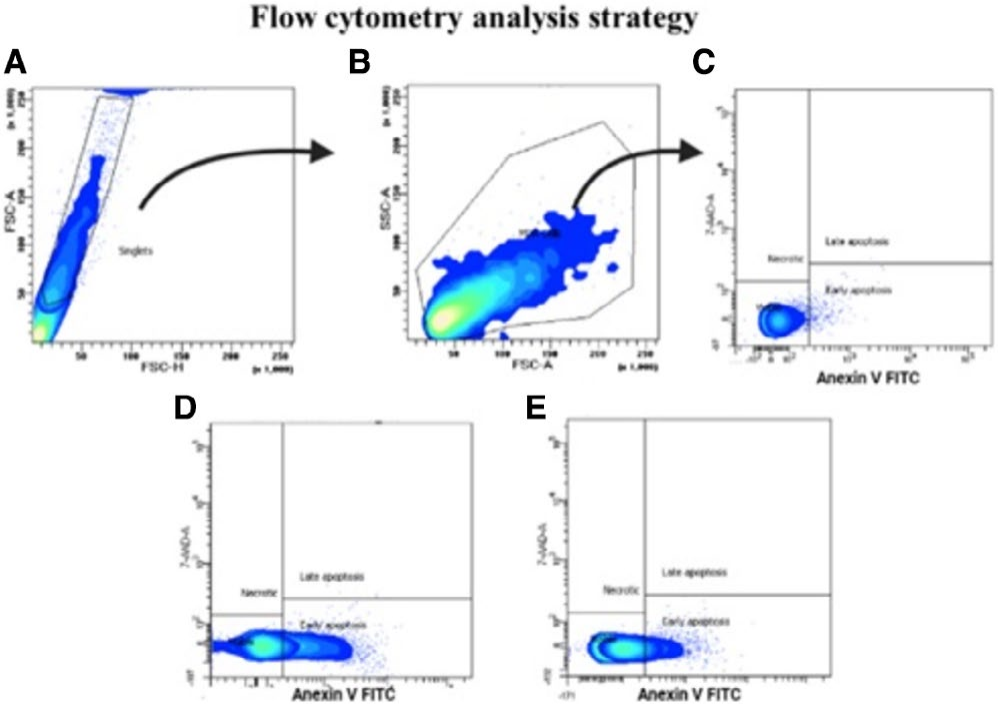
Flow Cytometry Analysis Strategy. After harvesting cells and performing flow cytometry staining, the analysis strategy was as follows: (A) Singlet events (to discard grouped cells); (B) Size (FSC) versus Granularity (SSC) dot plot to identify MDA‐MB‐231 cells; (C) Annexin V‐FITC versus 7AAD dot plot to identify live, necrotic, early, and late apoptotic cells. Negative control (DMSO); (D) Positive control (50 μM TSA); (E) The same analysis strategy was used for cells treated with different compounds. A total of 5000/seg events were analyzed for each group.

This work presents the apoptotic induction of the 2,6‐DKPs **1**, (*S*)‐stereoisomers **2a** to **6a** derived from Gly, Ala, Val, Leu, Ile, and Pro α‐amino acids, respectively; as well as **9a** to **14a** derived from Tyr, Tyr (where the OH group is protected with an acetamide group), Ser, Thr, Met, and Trp α‐amino acids, respectively on the MDA‐MB‐231 cell line by flow cytometry. In this assay, 1% DMSO was used as a negative control and TSA as a positive control (Figure 2). The inhibition of proliferation may be due, among other mechanisms, to arrest in the cell cycle, and/or to an induction of cell death. In the second case, it is important to differentiate whether this death is necrotic inflammatory or apoptotic non‐inflammatory, which is desirable for anti‐cancer agents. To distinguish between these types of death, we performed a flow cytometry experiment that distinguished between cell death with alteration in the plasma membrane, which allows the incorporation of 7AAD and is indicative of necrosis, and on the other hand, the expression of phosphatidyl serine in the membrane, which is characteristic of apoptotic bodies, Figure 5 (Li et al. 2023; He et al. 2014).

The results showed that all the compounds studied induced more apoptosis at 48 h than at 24 h in a range of 54.1 to 76.2%, except **12a** which exhibited a 3% decrease compared to the 24 h test. In addition, the percentage of cell death by necrosis was less than 5%. The data show that the cell death induced by the DKPs under study is via apoptosis more than necrosis, as indicated in Figure 2.

It was observed that 2,6‐DKPs **1**, **2a**‐**6a**, and **9a**‐**14a** induce more apoptosis at 48 h than at 24 h in a range of 54.1%–76.2%. Similar to our findings, other studies with DKP have shown that the proliferation inhibition of cancer cell lines was attributed to apoptotic events rather than necrosis. There have been reports of other compounds derived from DKPs that have similarly induced apoptosis in cancer cell lines, such as the epithelial type A549 (basal alveolar epithelium) and HeLa (cervical epithelium) cells after 48 h of treatment with DKPs (Xu et al. 2019). However, only one report has demonstrated apoptosis induction in MDA‐MB‐231 and MCF‐7 breast cancer cell lines. In this particular case, a piperazine derivative with a distinct chemical structure called epipolythiodioxopiperazines was used (Cribioli et al. 2022). The research involving epipolythiodioxopiperazines observed an increase in the mitochondrial pathway of apoptosis, characterized by elevated levels of activated caspase 8 and tBID. This increase led to enhanced mitochondrial permeability, activation of caspase 9, and subsequent cell death. On the other hand, studies on DKPs in epithelial cancer cell lines did not investigate the effect of DKPs on the expression of Bcl‐2, Bcl‐X, Bax, or caspases (Li et al. 2023; Cribioli et al. 2022). Therefore, our present work contributes to the understanding of the effect of 2,6‐DKP in inducing apoptosis in the MDA‐MB‐231 breast cancer cell line. However, further research is still needed to explore the intrinsic mechanisms underlying this apoptotic process.

## 3 Conclusions

We report herein the antiproliferative activity of twenty‐four 2,6‐diketopiperazines, **1** and the stereoisomers (*S*)‐**2**–**14**, and (*R*) **2**–**4**, **6**–**8**, **11**–**14** derived from α‐amino acids on the MDA‐MB‐231 cell line suggest that the effect depends on stereochemistry, as well as the type of substituent on the chiral center, and adopted conformations of the compounds under study. In addition, the apoptotic induction of the 2,6‐DKPs over the MDA‐MB‐231 cell line showed that all the compounds studied induced more apoptosis at 48 h than at 24 h in a range of 54.1%–76.2%, except **12a**, which exhibited a 3% decrease compared to the 24 h test. According to the biological assays, all the evaluated 2,6‐DKPs derivatives could be considered for further studies in vivo, principally **1**, **2a**, **4a**, and **5a** as possible anticancer agents. The compounds **1**, (*S*)‐**2a**, −**4a**, and ‐**5a** assayed on the Vero kidney cell line did not exhibit toxicity; this suggested that they are promising for in vivo assays soon.

## 4 Experimental Section

Cell lines of the MDA‐MB‐231 of TNBC and VERO from healthy kidney cells used in this work were donated by ‘National Institute of Cancerology of the Ministry of Health’ from Mexico. MDA‐MB‐231 and Vero cells preserved at −70°C in DMEM F12 medium Gibco, 10% FBS, 5% DMSO, and 5% glycerol were thawed at 37°C and resuspended in DMEM F12 medium, supplemented with 10% FBS and antibiotic (penicillin 100 UI/mL, streptomycin 100 μg/mL and amphotericin 250 μg/mL) and then centrifuged at 2000 rpm for 10 min. The supernatant was removed, and the cells were resuspended in DMEM F12 medium, supplemented with 10% FBS and antibiotic, seeded in 25 cm^2^ culture flasks Corning, and incubated at 37°C with 5% CO_2_ until at least 90% confluence was reached.

### 4.1 Antiproliferative Activity Evaluation of 2,6‐DKPs on MDA‐MB‐231 and VERO Cell Lines

The antiproliferative effect induced for compounds **1**, the stereoisomers (*S*)‐**2**–**14** and (*R*) **2**–**4**, **6**–**8**, **11**–**14** by the MTT assay using MDA‐MB‐231 cells, only the cytotoxicity of compounds **1**, (*S*)‐**2a**, ‐4a, and ‐**5a** was evaluated in cell line culture healthy Vero kidney cells since they were the compounds that showed the lowest IC_50_ in TNBC. The MTT assay was used to measure metabolically active cells. Cells were seeded into 96‐well microtiter plates Corning at 1.0 × 10 ^ 5^4^ cells/well in 100 μL of DMEM‐F12 medium supplemented with 10% FBS and antibiotic Gibco (penicillin 100 IU/mL, streptomycin 100 μg/mL and amphotericin 250 μg/mL). After reaching confluence, cells were treated for 24 h with compounds **1**, (*S*)‐**2**–**14** and (*R*)‐**2**–**4**, **6**–**8**, **11**–**14** stereoisomers, at concentrations of 0.000021, 0.00229, 0.0073, 0.0243, 0.081, 0.27, 0.9, 3.0, 10.0, and 12.0 mM at 1% DMSO, with compounds **1**, (*S*)**‐2a**, ‐4a, and **‐5a** at concentrations of 0.00066, 0.00229, 0.0073, 0.0243, 0.081, 0.27, 0.9, 3.0,10.0, and 12 mM at 1% DMSO, using DMSO 1% in DMEM F12 as the negative control and TSA as the positive control at 50 μM (Sanaei and Kavoosi 2020). Post‐treatment, cell viability was measured by the conventional MTT dye‐reduction assay. Thus, 20 μL of MTT reagent (5 mg/mL) in PBS 1X was added to each well and incubated at 37^o^C for 4 h. Viable cells with active mitochondria reduced the MTT to an insoluble purple formazan precipitate that was solubilized with the subsequent addition of 50 μL of DMSO. The formazan dye was measured spectrophotometrically using a microplate reader Molecular Devices. The cytotoxic impact of each treatment was quantified by determining cell viability as a percentage relative to untreated control cells, referred to as the percentage of control. This is calculated using the formula: [(A595 or A570 nm reading of treated cells)/(A595 or A570 nm reading of untreated cells)] × 100 (Mosmann 1983; Plumb 1999). All assays were performed in triplicate. Cell proliferation percentages relative to growth control cells were calculated, and inhibitory concentration 50 (IC_50_) values were calculated using a regression analysis of the concentration/antiproliferative activity ratio and analyzed using the statistical package prism (Graph‐Pad Software, San Diego, CA, USA).

### 4.2 Apoptotic Induction of 2,6‐DKPs on MDA‐MB‐231 Cell Line

Assays were performed using the commercial kit Annexin V labeled with the fluorochrome fluorescein isothiocyanate (FITC) BD Biosciences. Trichostatin A (TSA) Sigma was used as a positive control due to TSA demonstrating strong inhibitory effects against diverse cancer types through distinct pathways. Its anticancer potential has been observed in numerous in vitro and in vivo studies, encompassing various cell lines and animal models (Bouyahya et al. 2022). MDA‐MB‐231 cells were cultured and left until reaching 90% confluence as mentioned in the previous assay. Subsequently, the medium was removed and 100 μL of 2,6‐DCPs **1**, **2a‐6a**, and **9a‐14a** were added at their IC_50_ concentration in RMPI medium without FBS. The cells were incubated for 24 and 48 h at 37°C in a 5% CO_2_ atmosphere. Afterward, the supernatant was removed, and trypsinization of the adherent cells was performed by adding 300 μL of trypsin (Gibbco) and incubating at 37°C for 5 min. 300 μL of DMEM‐F12 supplemented with 10% FBS was added and centrifuged; the medium was decanted and 500 μL of 1X annexin V binding buffer was added to resuspend the cells. Subsequently, 100 μL of the suspension was placed in a 5 mL culture tube, and a mixture of 2.5 μL of Annexin V‐FITC and 1 μL of 7‐ADD was added to each cell suspension, incubated at 4°C for 10 min in the dark. After incubation time, the quantification of MDA‐MB‐231 cells was performed by flow cytometry with a FITC and 7AAD signal detector (FL1) and (FL3) on a FACS aria (Becton Dickinson). 5000/seg events were analyzed. Apoptotic induction percentages were calculated (Graph‐Pad Software, San Diego, CA, USA).

## Conflicts of Interest

The authors declare no conflicts of interest.

## Supporting information


**Data S1.** Supporting Information.

## Data Availability

The data that supports the findings of this study are available in the Supporting Information S1 of this article.
