# Supplementary material for: Selective Antiproliferative and Apoptotic Effects of 2,6‐Diketopiperazines on MDA‐MB‐231 Triple‐Negative Breast Cancer
Source: Chem Biol Drug Des. 2025 Apr 2;105(4):e70098. doi: 10.1111/cbdd.70098 (PMC11965977; doi:10.1111/cbdd.70098)
Supplement: Supplementary file 1 — Data S1. Supporting Information. [file CBDD-105-e70098-s001.docx]

Supplementary Information

Selective Antiproliferative and Apoptotic Effects of 2,6-Diketopiperazines on MDA-MB-231 Triple-Negative Breast Cancer

Flor Paulina Garrido González^1^, Elvia Mera Jiménez^2^, Martha Edith Macías Pérez^2^, Octavio Rodríguez Cortés^2^, Teresa Mancilla Percino^1,^*

^1^Centro de Investigación y de Estudios Avanzados del Instituto Politécnico Nacional, Departamento de Química, Apartado Postal 14-740, CP 07000, Ciudad de México, México.

^2^Instituto Politécnico Nacional, Sección de Estudios de Posgrado e Investigación, Escuela Superior de Medicina, Plan de San Luis y Diaz Mirón, CP 113400, Ciudad de México, México.

Graphical flow cytometry obtained from assay of the effect of 2,6-DKPs over MDA-MB-231 cell line at 24 h and 48 h.

**
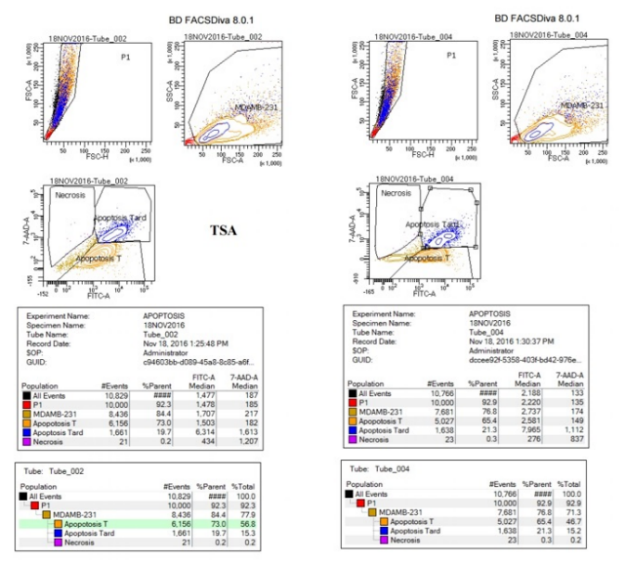

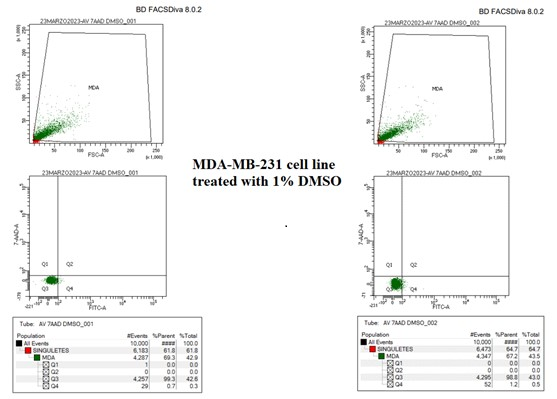
Graphical flow cytometry obtained at 24 h.**


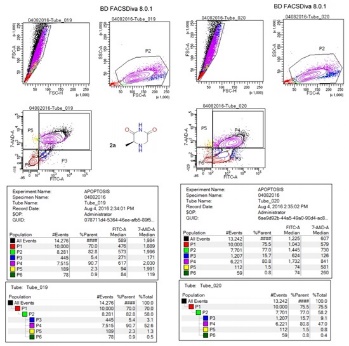

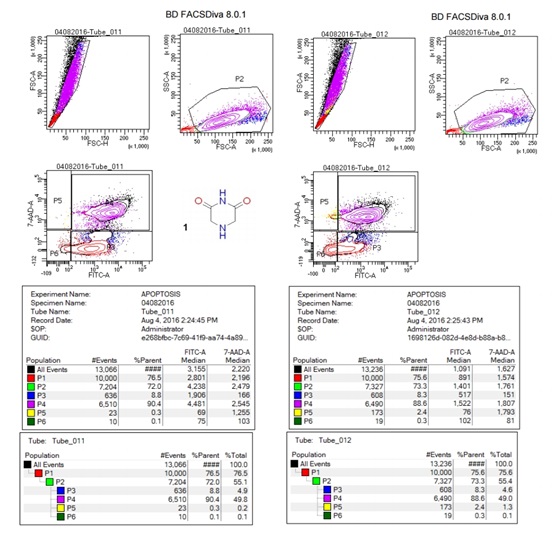


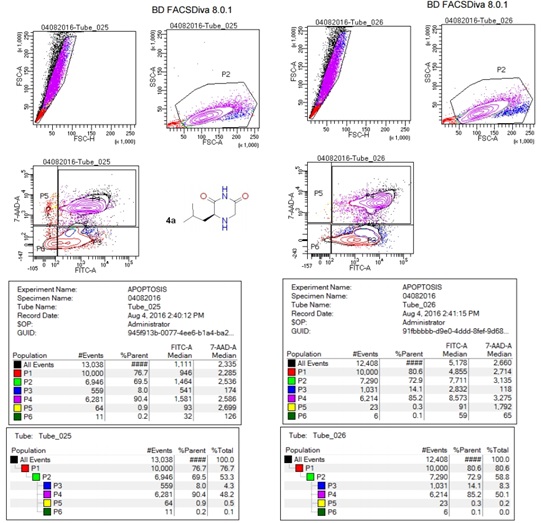

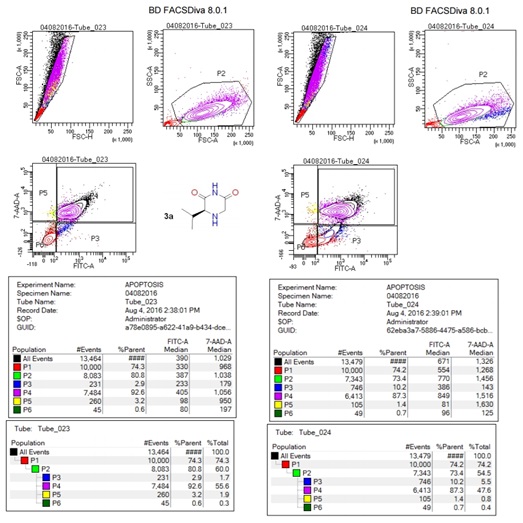


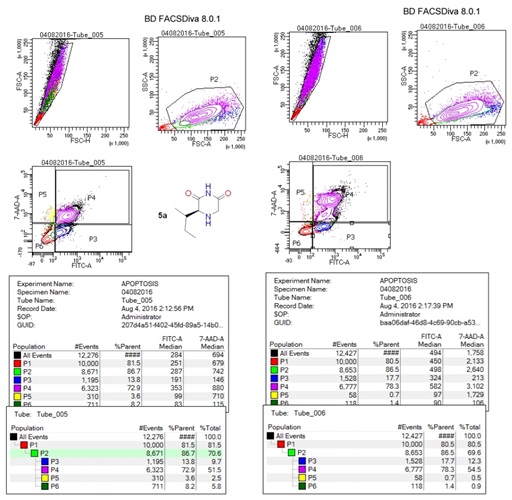

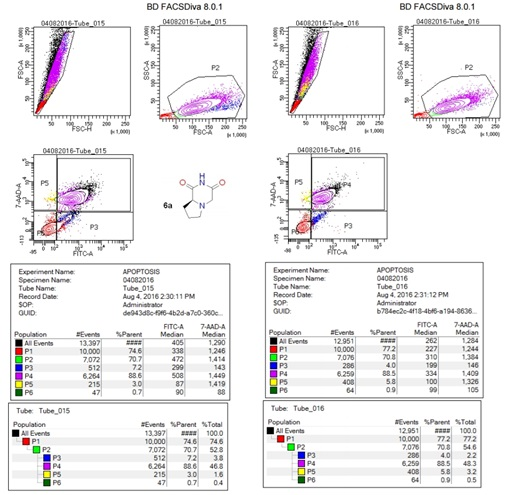


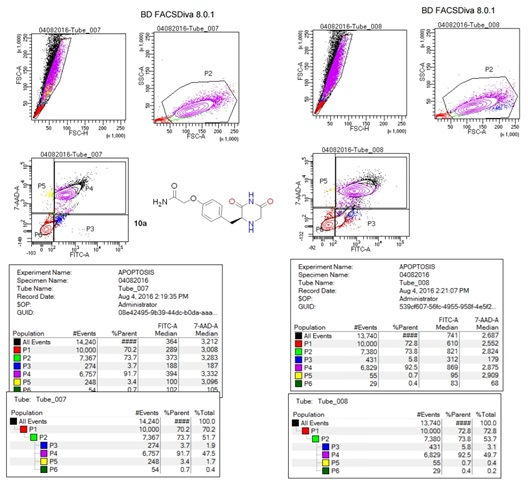

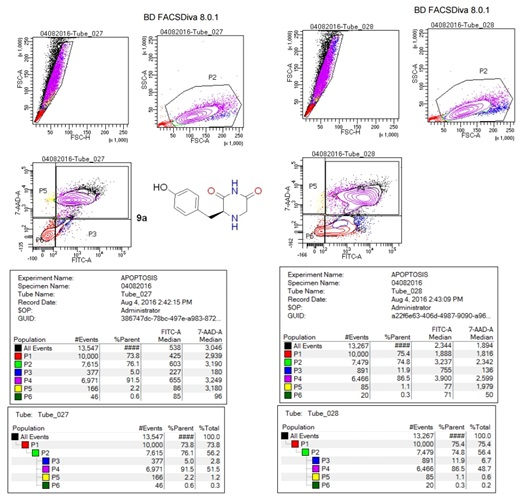


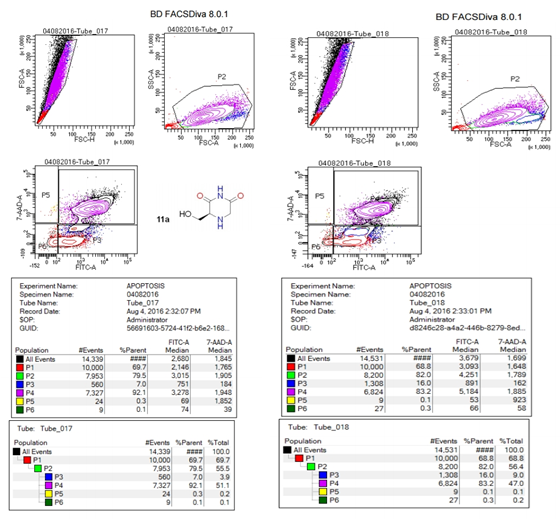

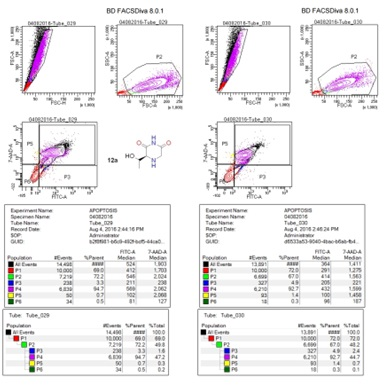


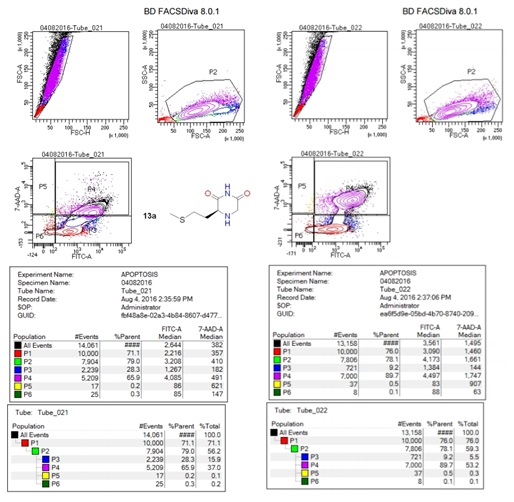

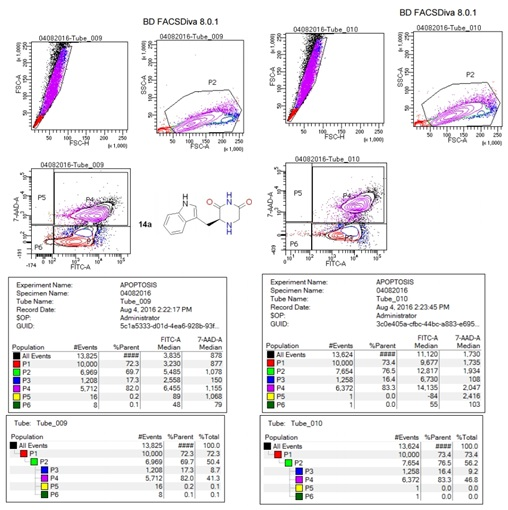


**Graphical of flow cytometry obtained at 48 h.**

**
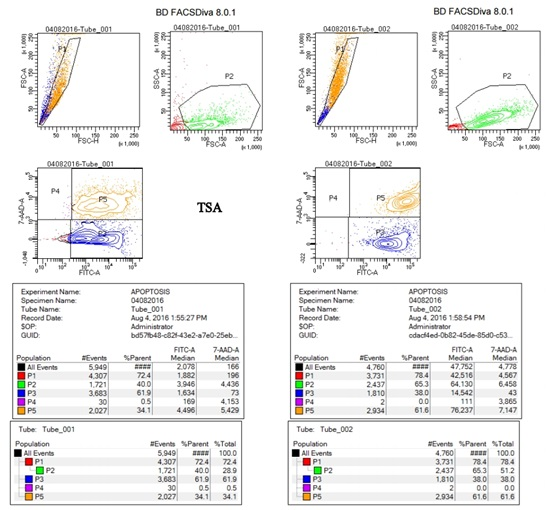

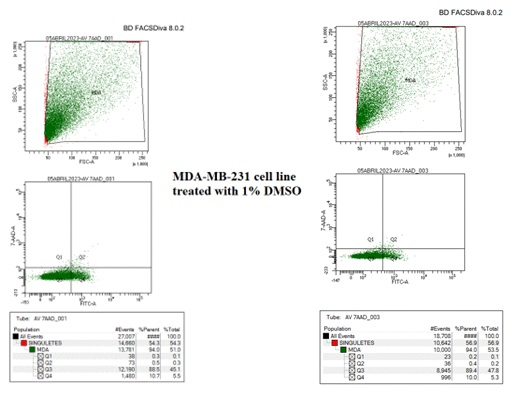
**

**
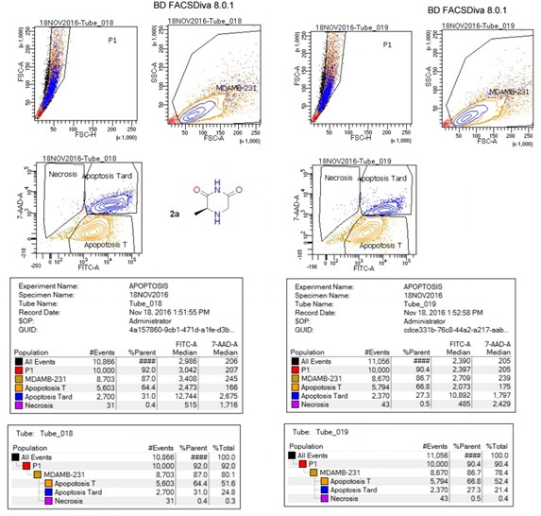

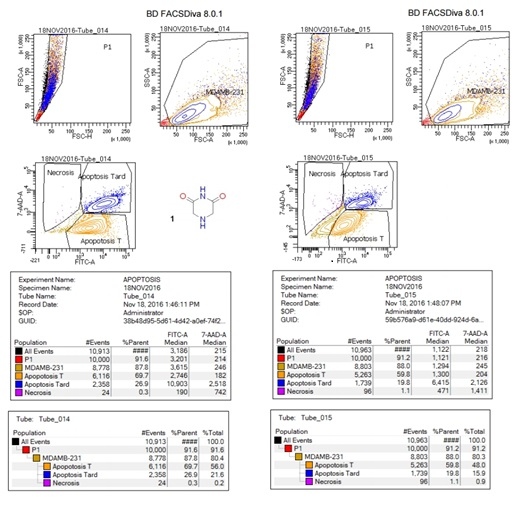
**

**
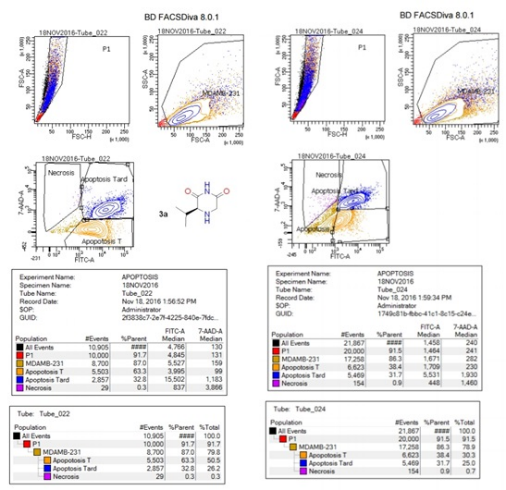

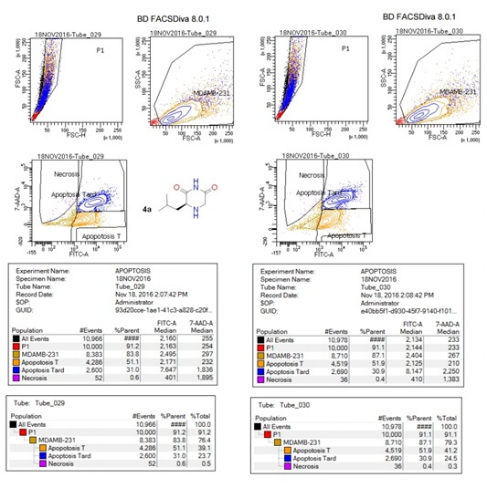
**

**
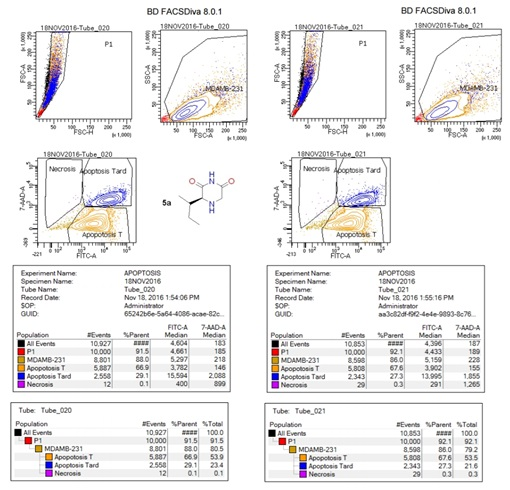

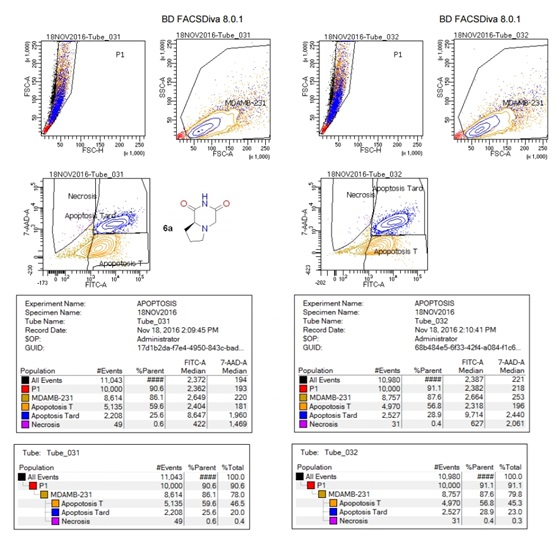
**

**
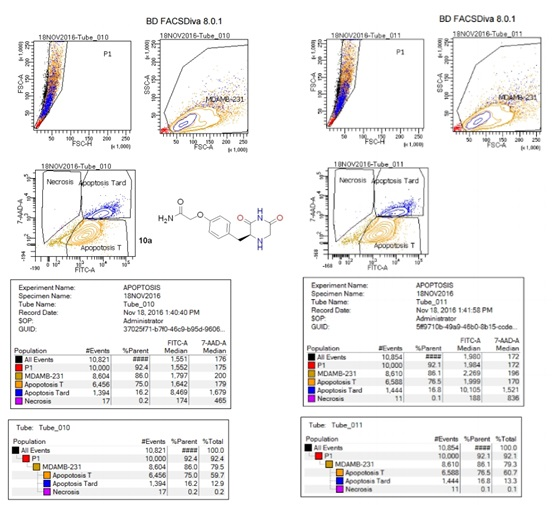

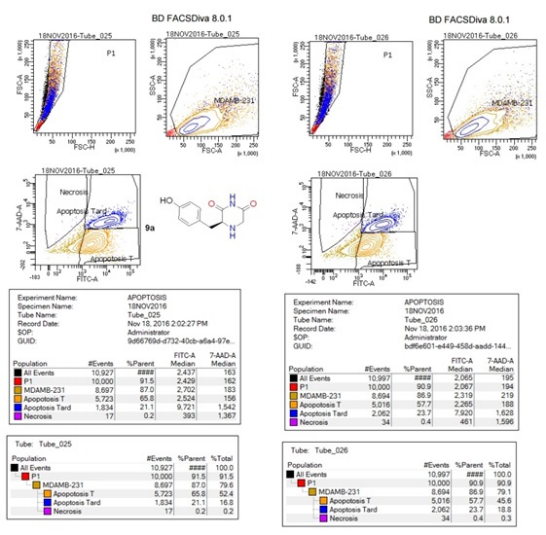
**


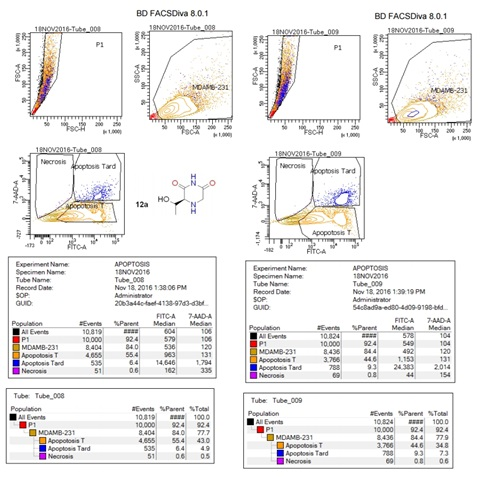


**
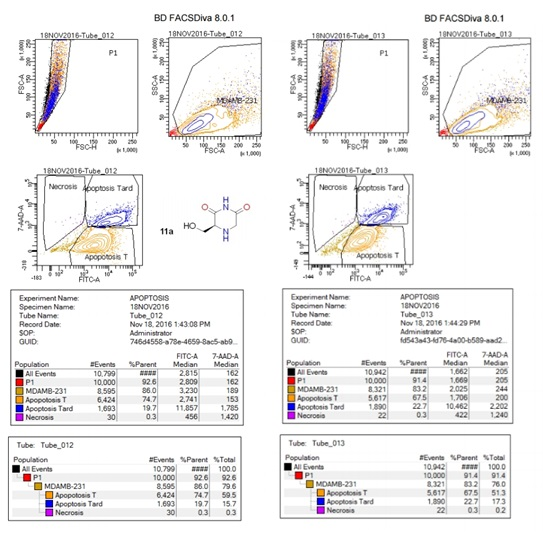
**


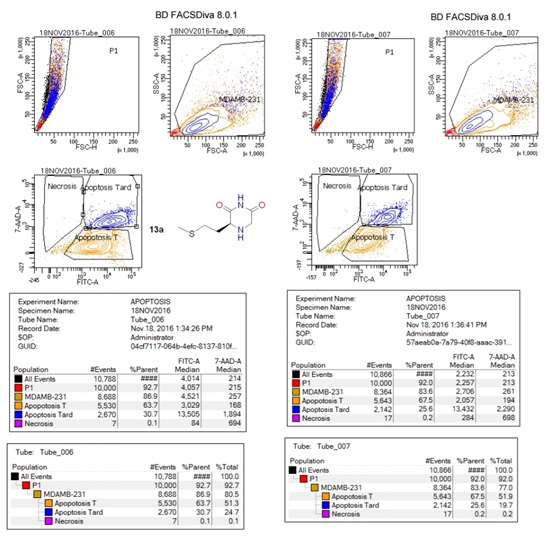

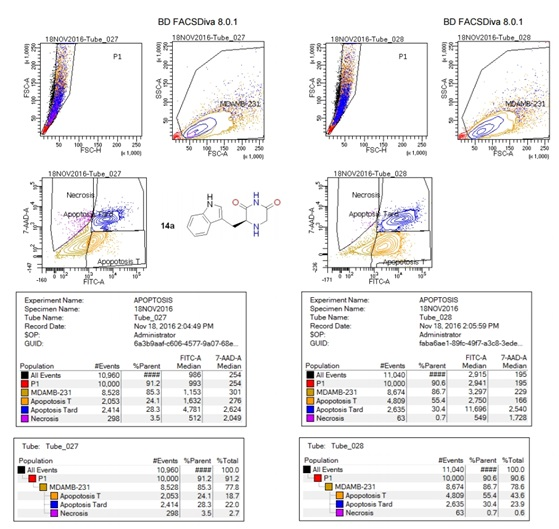


2-DKPs showed a preference for apoptotic cell death induction, as shown in Fig. 1.


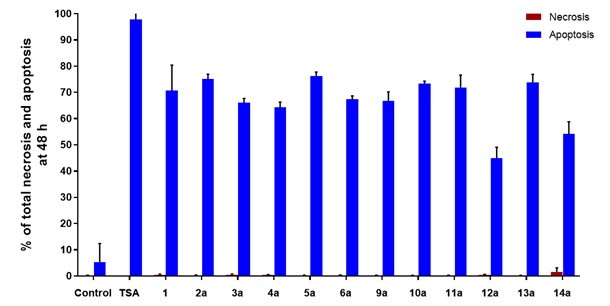


**Fig. 1**  Percentage of total necrosis and apoptosis cells of the MDA-MB-231 cell line treated with 1% DMSO (negative control), 50 µM TSA (positive control), and with the IC_50_ values of 2,6-DKPs **1**, **2a**-**6a** and **9a**-**14a** at 48 h.

Figure 2 shows concentration-response curves for 2,6-DKPs enantiomeric (*S*)- and (*R*)-pairs **12** and **13** derived from Thr and Met α-amino acids, respectively over the MDA-MB-231 cell line. (*S*) stereoisomers **12a** and **13a** were shown to be more potent than their corresponding (*R*) enantiomers.


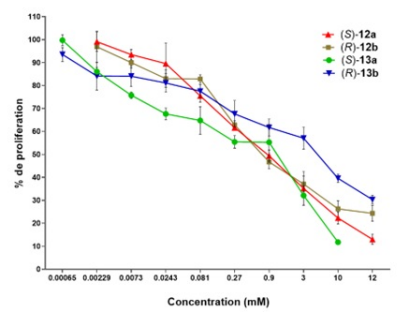


**Fig. 2** Concentration-response curves for 2,6-DKPs enantiomeric (*S*)- and (*R*)-pairs **12** and **13** derived of Thr and Met α-amino acids, respectively over the MDA-MB-231 cell line, n = 8, p < 0.0001.
